# A Framework for Locally Imputing and Predicting Biomarker Trajectories Under Irregular Monitoring: Application to Chronic Myeloid Leukemia

**DOI:** 10.21203/rs.3.rs-8420996/v1

**Published:** 2026-01-07

**Authors:** Felipe Montano-Campos, Patrick Heagerty, Eric Haupt, Erin Hahn, Jerald Radich, Aasthaa Bansal

**Affiliations:** University of Southern California; University of Washington; Kaiser Permanente; Kaiser Permanente; Fred Hutchinson Cancer Center; University of Washington

## Abstract

Irregular monitoring and missing data limit the utility of longitudinal biomarkers in real-world practice. We developed a generalizable framework that combines interval-aligned preprocessing, localized multiple imputation, and machine-learning forecasting to generate complete trajectories and predict future biomarker values under routine clinical conditions. Using BCR::ABL1 monitoring in chronic myeloid leukemia as a case study, we aligned measurements to 90-day intervals, applied a windowed, uncertainty-propagating imputation strategy, and trained recurrent neural network (RNN) and XGBoost models to forecast values three and six months ahead. Full Information models achieved RMSEs of 1.22–1.24 for 3-month predictions—well below the biomarker’s observed variability—and maintained accuracy even when the most recent visit was intentionally omitted, simulating extended follow-up. This framework preserves local temporal structure, supports individualized monitoring decisions, and is directly adaptable to other continuous biomarkers measured under irregular real-world schedules.

## Introduction

Longitudinal biomarkers are central to precision medicine, as changes in biomarker levels can guide monitoring strategies, indicate disease progression, and allow timely intervention, ultimately improving outcomes. In “real-world” practice, the clinical utility of biomarkers measured over time is often limited due to irregular patient monitoring and associated incomplete data. Patient adherence, financial barriers, anxiety about test results, and inconsistent follow-up contribute to missing data and irregular testing intervals, which limit data analysis attempting to leverage the longitudinal biomarker data to forecast future biomarker values and guide both individualized care and population-level modeling [1], [2]. To address this challenge, we developed a local imputation and prediction framework for continuous biomarkers collected under real-world conditions. Our approach draws on previous work by applying a local imputation strategy to resolve missing values and uses machine learning methods—Recurrent Neural Networks (RNNs) and Extreme Gradient Boosting (XGBoost)—to forecast future biomarker values [3], [4], [5]. Unlike traditional imputation strategies that model entire trajectories or assume smooth global trends, our method fits separate models within short, fixed-length windows, using neighboring biomarker values and patient characteristics to generate interval-specific imputations that preserve local variability and propagate uncertainty [6], [7].

Our framework is designed to preserve the variability of raw measurements, propagate uncertainty associated with irregular follow-up schedules through use of multiple imputations, and maintain predictive accuracy even when recent observations are skipped. We demonstrate the utility of this methodology in the context of chronic myeloid leukemia (CML), where longitudinal monitoring of BCR::ABL1 transcript levels guides treatment decisions. Current clinical guidelines recommend testing every three months after therapy initiation until major molecular response (≤ 0.1% International Standard (IS)) is achieved, followed by testing every three to six months [8]. However, these fixed intervals do not adapt to heterogeneity in relapse risk [9], [10], [11], and oncologists often face decisions about whether to shorten or extend the interval between assessments [12]. For patients showing stable responses, oncologists may extend the next monitoring interval to six months, effectively bypassing the next three-month BCR::ABL1 check. An analytic framework that can project future biomarker levels based on routine clinically captured biomarker histories can support such individualized scheduling decisions in a more data-driven manner. Therefore, we detail a comprehensive data science framework that combines the use of principled imputation to address irregular measurement together with machine learning methods to ultimately forecast a patient’s future biomarker distribution and guide decision making.

Using BCR::ABL1 monitoring in CML as a case study, we show how our proposed strategy can (i) generate complete longitudinal data, (ii) accurately forecast biomarker trajectories at three- and six-month horizons, and (iii) enable clinically relevant scenarios such as extending follow-up intervals for low-risk patients without loss of predictive accuracy.

## Results

The study cohort, summarized in Table 1, included 510 patients diagnosed with CML between 2007 and 2019. The mean age of the cohort was 54.6 years (SD 16.5), and the mean BMI was 29.4 (SD 6.6). The cohort was predominantly male (61.2%), with females comprising 38.8%. Regarding race and ethnicity, 43.5% of patients were Non-Hispanic White, 35.9% Hispanic, 9.8% Black, 9.0% Asian, and 1.8% identified as Other. Across all follow-up time points, the BCR::ABL1 values (included imputed measurements) ranged from − 8.29 to 1.08, with a mean of −4.29 (SD 2.83). The relatively high standard deviation reflects the inherent variability in the raw values. Unlike previous studies that often smooth BCR::ABL1 trajectories to reduce variability, we retained the raw measurements to preserve the richness of the data for our prediction analysis. This approach also enhances clinical applicability, as it aligns closely with the real-world data typically available in clinical settings, ensuring that our model predictions are directly translatable and implementable without requiring additional preprocessing. Quartiles spanned – 7.65 (1st quartile), −3.51 (median), and – 2.09 (3rd quartile), further emphasizing the broad range of values observed in the cohort.

**Figure 1:**
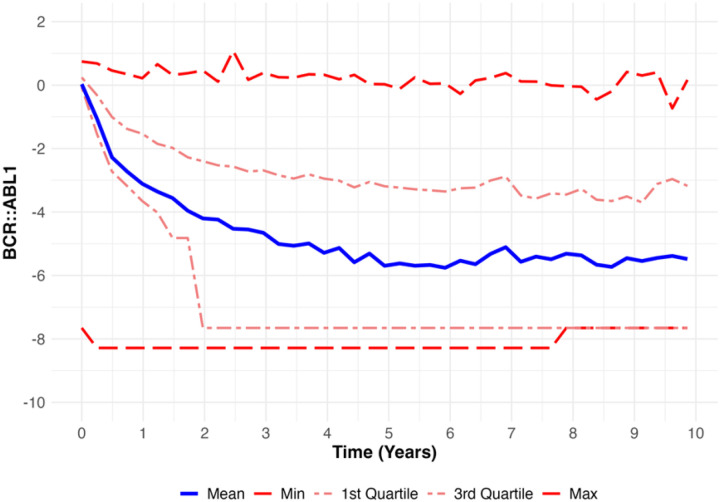
Longitudinal Summary of BCR::ABL1 Levels (Observed and Imputed) Across the Cohort

Figure 1 illustrates the longitudinal trajectory of BCR::ABL1 values across the cohort over a 10-year follow-up period (observed and imputed values). As anticipated, BCR::ABL1 values exhibit a sharp decline during the initial years following diagnosis, reflecting the early therapeutic response observed in most patients. This decline stabilizes over time, with mean values maintaining a sustained low level around – 5 in subsequent years. However, substantial variability persists throughout the follow-up period. The minimum BCR::ABL1 values remain consistently around – 8, while the maximum values reach approximately 1, underscoring the heterogeneity of patient trajectories. The interquartile range (IQR) remains around (−7.65, −2.09) across all time points after the first year of treatment initiation, emphasizing the continued spread of values.

The local imputation algorithm that fills in missing values using only nearby information (two leads ad two lags) demonstrated stable performance across iterations. Parameter estimates converged after approximately 15 imputation iterations, with the first lead (lead1) and lag (lag1) predictors consistently showing the strongest and most statistically significant contributions to the model. Their narrow confidence intervals and sustained predictive importance across all imputation periods indicate robust estimation of missing values. In contrast, the second lead term (lead2) was influential primarily during the first year following diagnosis, while the second lag term (lag2), though less prominent, remained statistically significant throughout follow-up (Appendix Figure A1). Visual inspection of imputed trajectories confirmed that the algorithm successfully preserved longitudinal trends, with imputed values closely following the direction and magnitude of observed measurements (Appendix Figure A2 and A3).

Table 2 shows RMSE values pooled across all 10 imputed datasets and summarized across all time periods to provide an evaluation of the predictive performance of the models. For the Full Information models (using all available prior BCR::ABL1 measurements, including the most recent visit) predicting *BCR*:: *ABL*1_*t*+1_, the RMSE was 1.24 (se = 0.039) for the RNN and 1.22 for XGBoost (se = 0.039). These RMSE values are well below the observed standard deviation of *BCR*:: *ABL*1 across the cohort (2.83), highlighting the models’ ability to explain variation and predict future values with precision relative to the inherent variability in the data. Given that *BCR*:: *ABL*1 values ranged from – 8.29 to 1.08, these errors represent a small fraction of the overall range and indicate that the models capture the underlying trajectory fairly accurately.

For the Skipped Last Visit models (withholding the most recent measurement to mimic an extended clinical interval) predicting *BCR*:: *ABL*1_*t*+1_, the RMSE increased to 1.33 (se = 0.032) for the RNN and 1.40 (se = 0.047) for XGBoost, reflecting a slight decline in predictive accuracy due to the exclusion of the most recent patient visit. Despite this reduction, the prediction errors remain relatively low, demonstrating the robustness of the models in handling scenarios with missing data. For predictions of *BCR*:: *ABL*1_*t*+2_, RMSE values increased for both Full Information and Skipped Last Visit models, as expected for a longer prediction horizon. Full Information models yielded RMSEs of 1.44 (se = 0.039) for the RNN and 1.37 (se = 0.032) for XGBoost, while the Skipped Last Visit models had RMSEs of 1.49 (se = 0.032) and 1.42 (se = 0.033) for RNN and XGBoost, respectively.

**Figure 2:**
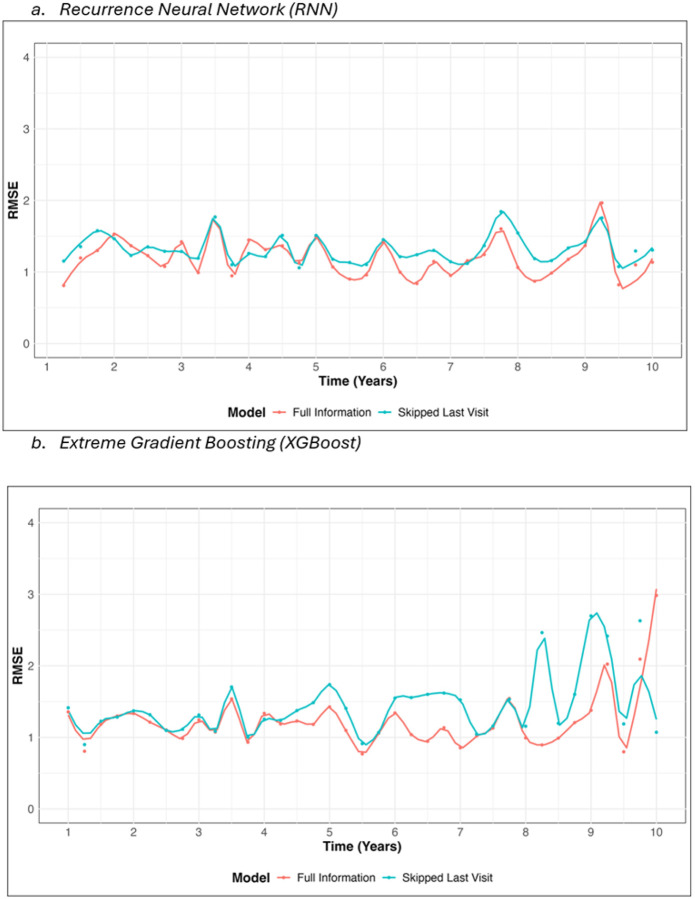
Root Mean Square Error (RMSE) of Predictive Models Over Time (t+1)

Figures 2a and 2b show the RMSE values for predicting *BCR*:: *ABL*1_*t*+1_ over a 10-year follow-up period. The RNN model (Fig. 2a) maintains consistent predictive accuracy, with RMSE values remaining stable around 1.2 across all time points for the Full Information model. Similarly, the Skipped Last Visit model performs consistently slightly worse but remains close to the RMSE of the Full Information predictions. The XGBoost model (Fig. 2b) displays a similar pattern, with RMSE values comparable to those of the RNN model up to year 8. However, after year 8, the RMSE for XGBoost predictions begins to show greater variability, particularly for the Skipped Last Visit model, indicating challenges in maintaining prediction consistency for longer follow-up periods. Both models perform well overall, but the RNN demonstrates more consistent accuracy throughout the entire follow-up period.

**Figure 3:**
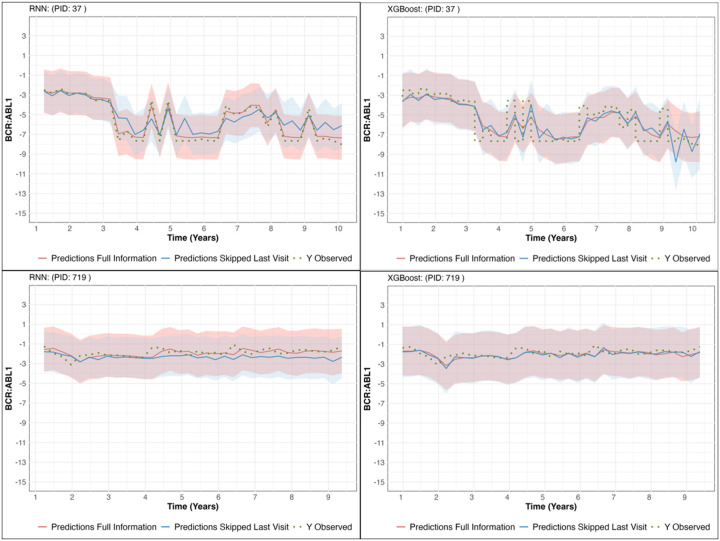
Observed Trajectories and Model Predictions of BCR∷ABL1_t+1_ with 95% Confidence Intervals for Two Random Test Set Patients

Finally, Fig. 3 illustrates the observed trajectories and model predictions for *BCR*:: *ABL*1_*t*+1_, along with 95% confidence intervals, for two randomly selected test set patients. Both the RNN and XGBoost models demonstrate strong alignment between predicted and observed values, with the Full Information model consistently providing tighter prediction intervals and closer adherence to the observed trajectories compared to the Skipped Last Visit model.

## Discussion

In this study, we introduced and validated a general framework for imputing and predicting longitudinal biomarkers under irregular monitoring conditions. We first applied a local imputation procedure to generate consistent longitudinal records, then used recurrent neural networks (RNNs) and extreme gradient boosting (XGBoost) models to forecast three- and six-month biomarker values with high accuracy. This approach preserves both the variability of raw measurements and the uncertainty propagated through multiple imputations. Importantly, it relies only on routinely collected clinical variables—such as biomarker levels, treatment adherence, and patient age—making it practical, scalable, and easily implementable in real-world settings without additional data collection. The framework is also generalizable to other continuous biomarkers beyond hematology, such as HbA1c, viral load, or prostate-specific antigen (PSA), where incomplete monitoring is common.

Applied to BCR::ABL1 monitoring in CML, the models demonstrated consistently strong performance, with RMSE values for the Full Information RNN model predicting *BCR*:: *ABL*1_*t*+1_ as low as 1.24 (SE = 0.039), well below the observed standard deviation of BCR::ABL1 across the cohort (SD = 2.83). This highlights the model’s ability to capture the inherent variability in the data with high precision. The Skipped Last Visit models exhibited higher RMSE values, such as 1.33 for the RNN and 1.40 for XGBoost at *BCR*:: *ABL*1_*t*+1_. These models simulate scenarios where low-risk patients are advised to extend the interval between follow-up visits, skipping the most recent biomarker collection. Despite this, the RMSE values remained within acceptable ranges and close to the overall variability of the data, indicating that the models could maintain a high level of predictive accuracy, even with this reduced information. Over a 10-year follow-up horizon, the RNN maintained stable error performance, while XGBoost displayed slightly increased RMSE variance beyond year 8, suggesting potential limitations in handling extended longitudinal sequences.

Most existing imputation methods for longitudinal biomedical data rely on global trajectory modeling or deterministic rules. Standard fully conditional specification (FCS) approaches, such as MICE, typically treat each time point as a separate variable but apply uniform model structures across the entire timeline, often overlooking temporal irregularity and abrupt short-term changes [19], [20]. Even extensions like two-fold FCS condition on adjacent timepoints but rely on global data cycling rather than fitting distinct, interval-specific models [20]. Other approaches—including trajectory-based means, interpolation, or hot-deck imputation—often impose smoothing assumptions or ignore residual variance [7]. In contrast, our approach fits a distinct linear model at each 90-day interval using localized information (two lead and two lag observations plus baseline covariates), then generates multiply imputed values by drawing from the model’s residual distribution. This interval-specific and stochastic imputation captures local temporal structure while allowing uncertainty propagation. Though related to moving-window frameworks like those proposed in [6], our design is uniquely aligned with guideline-based decision intervals in clinical practice, offering a localized, probabilistic alternative to globally structured strategies.

Prediction models based on BCR::ABL1 transcript levels have played a central role in optimizing CML treatment. Prior work has generally focused on estimating molecular response at predetermined time points. For instance, some studies applied robust and quantile regression to predict sustained deep molecular response (MR4.5) using BCR::ABL1 levels at months 3, 6, 9, and 12 of TKI therapy [21]. Others developed models to forecast BCR::ABL1 at 3, 6, and 12 months after treatment initiation [22]. While valuable, these approaches are bound to fixed schedules and do not adapt to the broader trajectory of an individual patient’s disease.

Our method, by contrast, enables continuous-time prediction of BCR::ABL1 over the next 3–6 months from any observed time point. This supports real-time, personalized monitoring rather than dependence on fixed milestones. Although most prior machine learning applications in CML have centered on classifying binary outcomes—such as relapse, treatment response, or diagnostic accuracy [23], [24], [25], [26], [27], our model predicts continuous BCR::ABL1 values directly. This provides fine-grained insights that can support binary classification tasks while retaining interpretability and flexibility.

Moreover, our framework is designed to support real-world clinical workflows, including scenarios where follow-up visits may be intentionally deferred for low-risk patients. Unlike models that assume regularly spaced observations, ours maintains predictive accuracy even when the most recent biomarker value is intentionally omitted—simulating a skipped visit. This capability allows the model to inform decisions about safely extending monitoring intervals without compromising risk assessment. In doing so, it contributes to ongoing efforts in precision oncology, complementing tools such as ensemble relapse predictors, deep learning-based diagnostic models, and architectures like CMLcGAN (AUC = 84.93%) [28], [29], [30], [31], [32], [33]. Taken together, our framework offers a clinically adaptive and data-efficient solution for longitudinal CML care.

While our methodology demonstrates high predictive accuracy and adaptability to real-world clinical settings, two limitations warrant consideration. Our adherence metrics, derived from pharmacy claims data, may not fully capture the timing or consistency of medication use. Additionally, the demographic and treatment characteristics of our cohort—drawn from an integrated healthcare system—may not reflect all CML patient populations, particularly those in other healthcare systems or geographic regions.

## Methods

We developed a methodological framework to address the challenge of incomplete longitudinal biomarker data under real-world monitoring conditions. The framework is designed to be generalizable to other disease settings with biomarkers that are collected irregularly in practice, such as HbA1c, viral load, or PSA. As a case study, we demonstrate application to BCR::ABL1 transcript monitoring in CML. We begin with a description of the CML case study, followed by details of the methodology used in the framework.

## Case Study

### Study Design and Data Source

We employed anonymized electronic health record data from Kaiser Permanente Southern California (KPSC), a large, integrated healthcare system serving approximately 4.5 million diverse members. The KPSC population is generally reflective of the broader Southern California population [13], [14]. The dataset included variables such as cancer diagnoses, patient demographics, BCR::ABL1 biomarker data, and detailed cancer treatment information, including pharmacy records. This study was approved by the University of Washington (UW) Institutional Review Board (Study #6191), with KPSC IRB relying on the UW IRB.

## Study Population

Our study cohort comprised patients diagnosed with CML between 2007 and 2019. Eligibility criteria required patients to be over 18 years of age and enrolled in the KPSC network at the time of diagnosis. We excluded patients who had only a single BCR::ABL1 measurement throughout their observation period and those not prescribed tyrosine kinase inhibitors (TKIs), the standard first-line therapy for CML that suppresses BCR::ABL1 activity and drives molecular response. Patients were followed until death, discontinuation of KPSC insurance, or the end of the study follow-up on May 18th, 2022, whichever occurred first.

### Biomaker Standardization

BCR::ABL1 is a critical biomarker for monitoring disease progression and therapeutic response in patients with CML. As the foundation of NCCN and ELN treatment guidelines, as well as a primary endpoint in phase 3 trials of second- and third-generation TKIs, peripheral blood BCR::ABL1 levels are integral to the clinical management of CML [8]. For this study, we utilized BCR::ABL1 measurements collected throughout each patient’s follow-up. Baseline values were defined as the measurement closest to the initiation of therapy following diagnosis. In cases where pre-treatment data were unavailable, the earliest measurement after treatment initiation was selected.

Given variations in the reporting of BCR::ABL1 levels—stemming from differences in measurement scales and formats across years—it was necessary to standardize all raw measurements. This was achieved using a log-ratio transformation, calculated as follows:

$$\text{BCR}::\text{ABL}1_{i,j}^{*}=\text{log}\left(\frac{\text{BCR}::\text{ABL}1_{i,j}}{\text{Median}\left(\text{BCR}::\text{ABL}1_{i,0}\right)}\right)$$

where *BCR*:: *ABL*1_*i*,*j*_ and *BCR*:: $\text{ABL}1_{i,j}^{*}$ denote the measured and standardized values, respectively, for individual i at time j. *BCR*:: *ABL*1_*i*,0_ represents the baseline value for individual i and the median was calculated over baseline values for all individuals measured using the same format as individual i. This standardization ensured consistency and comparability of BCR::ABL1 values across the cohort, regardless of the original measurement method. Standardized BCR::ABL1 values are critical not only for ensuring comparability across patients but also for contextualizing clinical interpretations. For example, peripheral blood BCR::ABL1 mRNA levels below 0.1% on the International Scale are associated with sustained remission and a very low risk or progression, while levels above this threshold indicate are associated with higher relapse and progression rates. These clinically established thresholds highlight the importance of accurate standardization in evaluating disease progression and treatment response.

## Calculation of Adherence Trajectory

We quantified treatment adherence in 90-day intervals by employing the continuous multiple-interval measures of medication availability/gaps (CMA9) metric, which calculates adherence trajectories as a function of drug dispensation dates, duration of supply, and amount dispensed [15]. Adherence at time t (*Adherence*_*t*_) represents adherence over the past three months, while *Adherence*_*t*−1_ reflects adherence from the prior three to six months. Adherence values ranged from 0 (no adherence) to 1 (perfect adherence), providing a continuous adherence trajectory for each patient.

## Imputation and Prediction Framework

The framework consists of four key steps in order to generate biomarker predictions for any subject:

- Step 1: Align observed biomarker measurements to clinically meaningful time intervals such as every 3 months or every 6 months,
- Step 2: Apply a multiple imputation strategy to fill in missing values that uses measurements locally within a specified time window and which preserves variability due to imputation of missing values,
- Step 3: Predict biomarker values three- and six-months in the future using machine learning models based on past biomarker trajectories and any other potential prognostic covariates, and
- Step 4: Evaluate predictive accuracy, pooled across multiple imputations.

Below we describe each step and its application to the case study in further detail.

### Step 1: Alignment of Observed Biomaker Measurements to Clinically Meaningful Regular Intervals

Generating data in consistent 90-day (three-month) intervals enables comparable data across patients. With this goal, we first leveraged observed biomarker data and aligned it to 90-day intervals, consistent with clinical guideline-based schedules[16].

We established a baseline date using the BCR::ABL1 collection closest to the time of diagnosis (time 0) and constructed expected collection dates at 90-day intervals from diagnosis to the final observed date. Each interval was paired with the nearest available observed measurement within a 45-day window; intervals without a nearby collection were marked as missing. This pre-processing ensured a standardized data structure across patients, aligning with clinical guidelines while addressing variations in monitoring frequency.

There are multiple pragmatic and clinical benefits of this standardized approach to measurement. First, consistent 90-day (3-month) intervals enable robust longitudinal analysis, ensuring reliable data over time. Second, these intervals support personalized monitoring by aligning with clinical guidelines and allowing adaptations based on individual patient characteristics and treatment responses. Finally, they facilitate accurate predictions by providing a structured framework for modeling future BCR::ABL1 levels using past data trends that can account for uncertainty due to any missing data imputation.

### Step 2: Local Imputation Algorithm

To address missing data within the aligned 90-day intervals, we implemented a local, windowed multiple-imputation algorithm that leverages information from adjacent biomarker values, patient demographics, and treatment characteristics. The algorithm is designed to preserve local temporal structure and variability in the biomarker trajectory.

To impute a measurement for patient *i*, at a time point *t*, denoted as *Y*_*it*_, we defined

$$Z_{i,t}=\left[Y_{i,t-2},Y_{i,t-1},Y_{i,t+1},Y_{i,t+2}\right]$$

as the vector of two lagged and two lead biomarker values, after de-meaning each variable across the cohort to center it at zero. Here we consider two previous and two future observations but alternative window selection is possible. We further defined *X*_*i*_ as the variables related to patients’ sociodemographic characteristics, including BMI, gender, age, and first-line treatment.

The following local linear model was fit using patients with non-missing data at time *t*:

$$Y_{i,t}=\alpha_{t}+\beta_{t}Z_{i,t}+\gamma_{t}X_{i}+\in$$

In cases where the initial iteration encountered missing lead and lag predictors, we imputed these with the overall mean of the variable (effectively a value of 0, since the columns were de-meaned). Using the trained model, we predicted ${\hat{Y}}_{i,t}$ for patients with missing values at that collection point within the patient’s follow-up time window. This prediction involved adding a normally distributed random draw (mean 0 and variance equivalent to the fitted model’s error term variance) to incorporate random variability. Alternative approaches such as predictive mean matching can be adopted to relax distributional assumptions and match the outcome support for any measurement.[17] We repeated this process for the next 90-day interval (3-month) data point, with each iteration building upon the imputed values from previous cycles. This iterative process was repeated 15 times (burn-in phase), with each cycle refining the imputed values based on previous iterations.^1^

To capture overall uncertainty, we generated 10 multiply imputed datasets after the burn-in phase to ensure stability. Details of the validation analyses, including algorithm stabilization, visual inspection of imputed trajectories, and performance using artificially introduced missing values, are provided in the Appendix.

### Step 3: Development of Prediction Models

We aimed to predict the level of BCR::ABL1 at three months and six months in the future (denoted *BCR*:: *ABL*1_*t*+1_ and *BCR*:: *ABL*1_*t*+2_, respectively) using past *BCR*:: *ABL*1 measurements and other patient characteristics. First, we split each of the 10 imputed datasets at the patient level into 80% training and 20% test sets. Each model was independently trained and tested across the 10 imputed datasets, and the 10 sets of results were combined to generate the final predictions (discussed further in next section).

In the training set, we fit two machine learning algorithms: Recurrent Neural Networks (RNNs) and Extreme Gradient Boosting (XGBoost). The RNN architecture is particularly suited for time series and sequential data. Unlike traditional feedforward networks that treat data points as independent, RNNs are designed to handle data with dependencies among consecutive elements, which is characteristic of longitudinal data like ours. RNNs incorporate memory to retain past information for predicting future elements in the sequence, enabling the network to understand context and dependencies within the sequence [3], [4].

XGBoost is a powerful and efficient implementation of gradient boosting that combines multiple weak learners, typically decision trees, to create a strong predictive model. Although XGBoost assumes that data points are independent, it has demonstrated good performance in various predictive scenarios, including time series forecasting, due to its robustness and prevention of overfitting through regularization. Its parallel processing capabilities also contribute to its efficiency [5].

Both algorithms were applied across two model specifications: the **Full Information model** and the **Skipped Last Visit model.** The Full Information model included the current biomarker level (*BCR*:: *ABL*1_*t*_), the biomarker levels from three and six months prior (*BCR*:: *ABL*1_*t*−1_ and *BCR*:: *ABL*1_*t*−2_), TKI treatment adherence at the current visit (*Adherence*_*t*_) and the previous visit (*Adherence*_*t*−1_), age at diagnosis, and time since diagnosis. In contrast, the Skipped Last Visit model excluded the biomarker level from three months prior (*BCR*:: *ABL*1_*t*−1_) and incorporated the current biomarker level (*BCR*:: *ABL*1_*t*_), the six-month BCR::ABL1 change, defined as the difference in biomarker levels between six months ago and today, TKI treatment adherence at the current visit (*Adherence*_*t*_) and the prior visit (*Adherence*_*t*−1_), age at diagnosis, and time since diagnosis.

The Skipped Last Visit model may be particularly useful in scenarios where oncologists decide to extend the monitoring interval for low-risk patients, thus using a biomarker trajectory with one skipped visit to predict future biomarker levels. We fit separate models for predicting future biomarker levels *BCR*:: *ABL*1_*t*+1_ and *BCR*:: *ABL*1_*t*+2_, enabling continuous patient monitoring and therapy adjustment.

### Step 4: Evaluation of Predictive Accuracy

To assess model performance, we utilized root mean square error (RMSE) as the primary evaluation metric. As mentioned above, each model was independently trained and tested across the 10 imputed datasets. Predictions from the ten imputed datasets were consolidated using Rubin’s rules, which combine results across multiple imputations by accounting for both within-dataset variability (the uncertainty of predictions estimated within each imputed dataset) and between-dataset variability (the uncertainty arising from differences across imputations). This method enabled us to derive a combined prediction and its standard error, which was subsequently used to compute confidence intervals [18]. RMSE values, along with their associated standard errors, were calculated to evaluate model performance. Results are presented both as aggregate RMSE across all time periods and as RMSE for specific time points, offering an understanding of predictive accuracy over the entire follow-up period.

## Supplementary Material

Supplementary Files

This is a list of supplementary files associated with this preprint. Click to download.

- Appendix.docx

## Figures and Tables

**Table 1:** Baseline Demographic and Clinical Characteristics of the Study Cohort

Variable	Overall Cohort (N = 510)
Age (Mean (SD))	54.61 (16.48)
BMI (Mean (SD))	29.37 (6.55)
Sex, n(%)	
• Female	198 (38.82%)
• Male	312 (61.18%)
Race/Ethnicity, n(%)	
• Asian	46 (9.02%)
• Black	50 (9.8%)
• Hispanic	183 (35.88%)
• Non-Hispanic White	222 (43.53%)
• Other	9 (1.76%)
Smoking Behavior, n(%)	
• Never	302 (59.22%)
• Quit	166 (32.55%)
• Yes	39 (7.65%)
Baseline Treatment, n(%)	
• Imatinib	409 (80.2%)
• Dasatinib	82 (16.08%)
• Nilotinib	19 (3.73%)
Year of diagnosis, n(%)	
• 2007–2010	90 (17.65%)
• 2011–2014	143 (28.04%)
• 2015–2019	277 (54.31%)
BCR::ABL1 (Across Follow up)	
• Mean (SD)	−4.29 (2.83)
• Median	−3.51
• IQR	(−7.65, −2.09)
• (Min, Max)	(−8.29, 1.08)

**Table 2:** Root Mean Square Error (RMSE) with Associated Standard Errors for Prediction Models Evaluated Across All Time Points.

	RNN	XGBoost
Full Information		
• *BCR*: :*ABL*1_*t*+1_	1.24 (0.039)	1.22 (0.039)
• *BCR*: :*ABL*1_*t*+2_	1.44 (0.039)	1.37 (0.032)
Skipped Last Visit		
• *BCR*: :*ABL*1_*t*+1_	1.33 (0.032)	1.40 (0.047)
• *BCR*: :*ABL*1_*t*+2_	1.49 (0.032)	1.42 (0.033)

## Data Availability

The data that support the findings of this study are available from Kaiser Permanente Southern California (KPSC) but restrictions apply to the availability of these data, which were used under license for the current study and are therefore not publicly available.

## Footnotes

1. **Special Considerations for Initial Data Points:** It is worth noting that our algorithm’s subsequent iterations built upon previously imputed values, progressively filling in initial gaps. Therefore, the imputation of lead and lag **predictors** occurred only during the first pass of the algorithm. To address the absence of preceding biomarker data at initial collection points: **1) Time 0 (Baseline):** Since no prior collections exist, the model included only the data from the two subsequent (lead) collection points. **2) First Three-Month Interval (90 days):** At this point, the model used one preceding (lag) collection and the two subsequent (lead) collection points as predictors.

## Code Availability

The custom code used to preprocess the data, implement the imputation procedures, and train the prediction models is not publicly available due to data-use restrictions. However, the code may be made available to qualified researchers upon reasonable request to the corresponding author.
